# Portable endovascular disposable robotic assistant (PEDRA) for image-guided peripheral endovascular interventions: technical feasibility, safety, and future implications

**DOI:** 10.1186/s42155-026-00789-2

**Published:** 2026-11-14

**Authors:** Joe Kevin Khoury, Nicholas Joseph Lima, Ahmed Elsahhar, Hanzhou Li, John Timothy Moon, Amir Pourmorteza, David Prologo, Junman Kim, Zachary Bercu

**Affiliations:** Department of Radiology and Imaging Sciences, Emory University School of Medicine, Atlanta, GA USA

**Keywords:** PEDRA, Robotic-assisted, Endovascular, Interventional radiology

## Abstract

**Background:**

Traditional fixed-console endovascular robots face barriers due to high capital costs and infrastructure requirements. This study evaluates the technical feasibility and safety of the LIBERTY® Endovascular Robotic System—a portable, disposable platform—across diverse Interventional Radiology procedures and vascular access sites.

**Materials and methods:**

A single-center retrospective review was conducted on 20 consecutive outpatient procedures performed by five operators. Cases included prostate artery embolization, uterine fibroid embolization, renal/gonadal venography, geniculate artery embolization, Y90 mapping, and Y90 radioembolization. Vascular access points included left radial and femoral approaches.

**Results:**

Procedural technical success was achieved in 100% (20/20) of cases. 100% of vessels attempted with robotic assistance were successfully selected (31/31). Complete robotic target-vessel selection—performing all procedural aspects of vessel selection robotically—was 75% (15/20). Partial robotic target-vessel selection—conversion to manual prior to completion of all vessel selections with robotic assistance—was 25% (5/20). All 5 cases of partial robotic target-vessel selection were due to hardware malfunction, rather than an inability to select target vessels with robotic assistance. Subgroup analysis of the last 10 cases after switching to utilizing updated units demonstrated 100% complete robotic target-vessel selection (10/10). No device-related serious adverse events or complications occurred.

**Conclusion:**

The portable and disposable robotic platform was successfully integrated into a diverse Interventional Radiology practice, demonstrating the first documented prostate artery embolization, uterine fibroid embolization, renal/gonadal venography, geniculate artery embolization, and Y90 procedures by portable and disposable robotic-assistance, as well as the first robotic-assisted endovascular procedures via radial access. Its design may lower economic and institutional barriers, potentially expanding robotic-assisted interventions to rural or non-academic settings. The portable robotic assistant is safe and feasible for complex endovascular interventions. Further prospective studies are needed to quantify ergonomic benefits and radiation reduction.

**Supplementary Information:**

The online version contains supplementary material available at https://doi.org/10.1186/s42155-026-00789-2.

## Manuscript

### Background

Fluoroscopically guided procedures span multiple procedural and surgical specialties in medicine and expose operators to radiation and ergonomic strain despite modern shielding and dose-reduction technology. Among interventional radiologists, as much as 88% of physicians experience musculoskeletal pain and up to 21.2% of these physicians were unable to work over the same period [1]. In the context of these occupational hazards, endovascular robotic platforms emerged as a structural solution designed to decouple catheter manipulation from operator proximity to the radiation field. Over the last two decades, multiple console-based endovascular robotic platforms have emerged including Corpath (Corindus Vascular Robotics), the Magellan System (Hansen Medical), and the R-One (Robocath), all of which demonstrated technical feasibility and radiation reduction of primary operators [2–7]. However, the high upfront capital equipment costs and required infrastructure dependencies have limited adoption of these technologies to specialized centers of care.

**Fig. 1:**
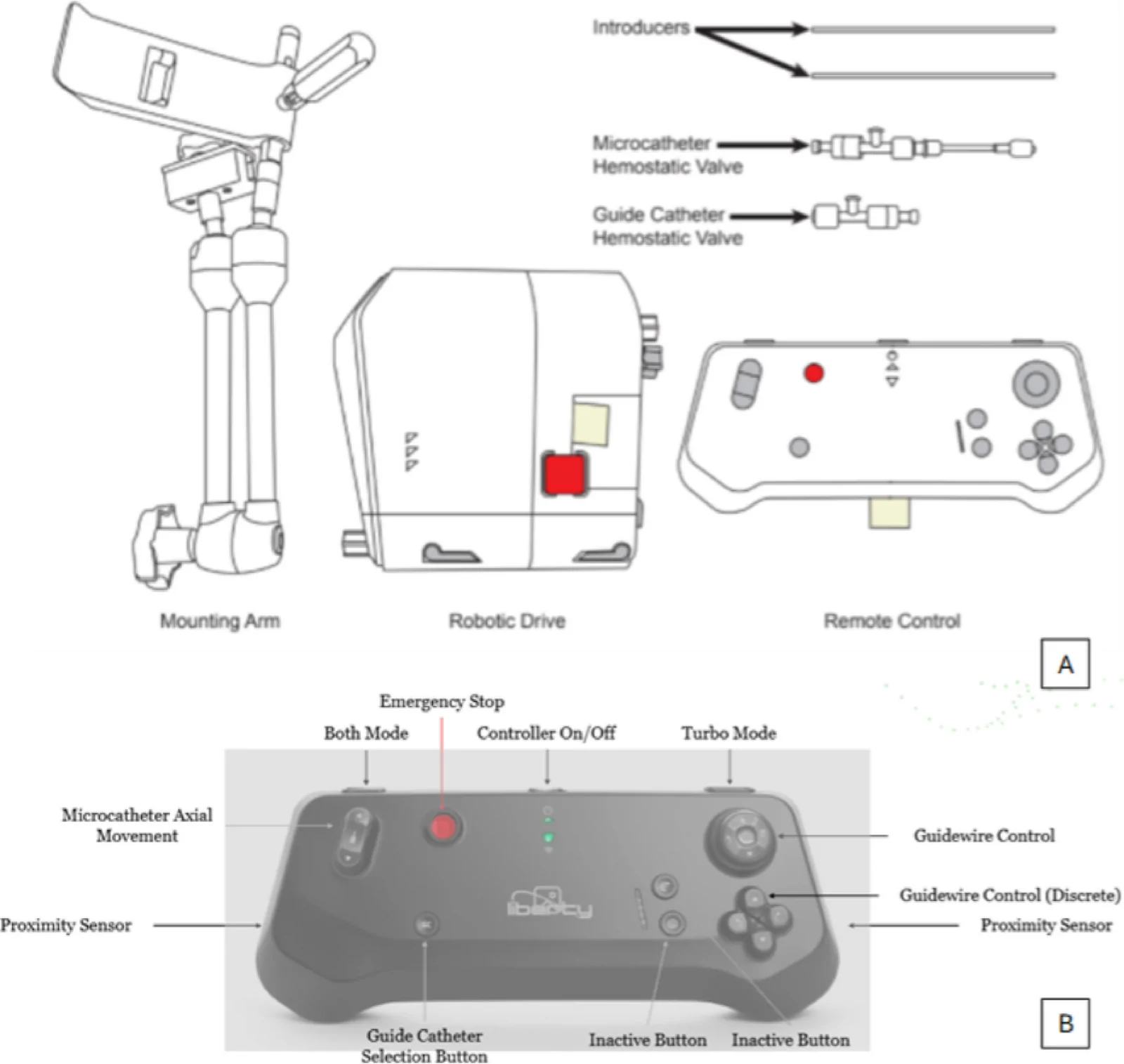
**A** LIBERTY® Endovascular Robotic System components (images courtesy of Microbot Medical, Hingham, MA, USA). **B** Labeled components of the LIBERTY controller

The translational challenge, therefore, is no longer whether robotic catheter manipulation is feasible, but whether it can be implemented in a manner that reduces infrastructural burden while preserving procedural flexibility. Recently, a portable, disposable endovascular robotic platform (LIBERTY® Endovascular Robotic System, Hingham, MA, USA) received regulatory clearance, representing a distinct departure from prior fixed-console systems. Figure 1 shows the layout of this robotic system, labeling the components that come in the packaging, as well as the buttons on the remote control. Whether such an architecture can be easily integrated across diverse interventional radiology procedures and access strategies has not been systematically evaluated.

We report the first clinical experience following regulatory clearance of a portable endovascular disposable robotic assistant (PEDRA), specifically the LIBERTY® by Microbot, in a series of consecutive image-guided interventions performed in real-world conditions with multiple operators, representing procedures from different access points and procedures not yet described in the literature, to create a point of reference for future proceduralists. We describe wire and catheter selection, compatibility, access site versatility with left radial and bilateral femoral approaches, technical success as it relates to target-vessel catheterization, and device-related safety outcomes.

## Materials and methods

This study was a single-center retrospective review of outpatient endovascular procedures performed by 5 total operators, with postgraduate experience ranging from 2 to 10 years. None of the operators had prior experience with performing endovascular robotic procedures. Most procedures were performed by a single operator, although select early cases involved two operators during initial adoption of the robotic system. Institutional Review Board (IRB) approval was obtained. Patient consent for procedural data usage was obtained. This study was not industry sponsored. All of the utilized robotic devices were purchased without an early adoption arrangement. There are no relevant relationships with the manufacturer. The cases performed with utilization of the PEDRA spanned liver-directed therapies, genitourinary and musculoskeletal interventions on a rolling basis, based on upcoming outpatient endovascular procedures. During the informed consent process, patients were given the option to have the procedure done with or without robotic assistance. The procedures performed with PEDRA included prostate artery embolization (PAE) procedures, uterine fibroid embolization (UFE), renal/gonadal venography, geniculate artery embolization (GAE) procedures, selective hepatic angiography procedures for Y90 mapping, and Y90 radioembolization of a hepatic tumor. The access points for the procedure were versatile, including left radial artery, right femoral artery, right femoral vein, and left femoral artery.

Procedural technical success was defined by the completion of intended treatment. For PAE, this included embolization of unilateral or bilateral prostatic arteries depending on anatomic favorability. For UFE, this included successful bilateral fibroid embolization. For renal/gonadal venography, this meant successful venography of both the left renal and gonadal veins. For GAE, this included selecting and treating at least 2 geniculate arteries that demonstrated hyperemia on angiographic evaluation. For hepatic mapping angiography, this included successful planning angiography prior to future planned directed liver therapy. For the Y90 radioembolization, this included successful delivery of Y90 to the hepatic tumor as planned.

Complete robotic target-vessel selection was defined by completion of the procedure with selection of *all* target vessels to complete the procedure with robotic assistance. If one target vessel was selected and treated with robotic assistance, but a different target vessel was subsequently selected manually, this was considered partial robotic target-vessel selection. Partial robotic target-vessel selection was subdivided into two groups: unsuccessful robotic selection and robotic malfunction.

Total robotic attempted vessels selected describes the total number of vessels that were successfully selected out of all attempts with robotic assistance over the course of the 20 cases, which often was more than one vessel per procedure. This most accurately reflects the capability of robotic assistance for vessel selection.

Procedure time was measured from the time of the initial scout obtained to the last acquired image.

**Fig. 2:**
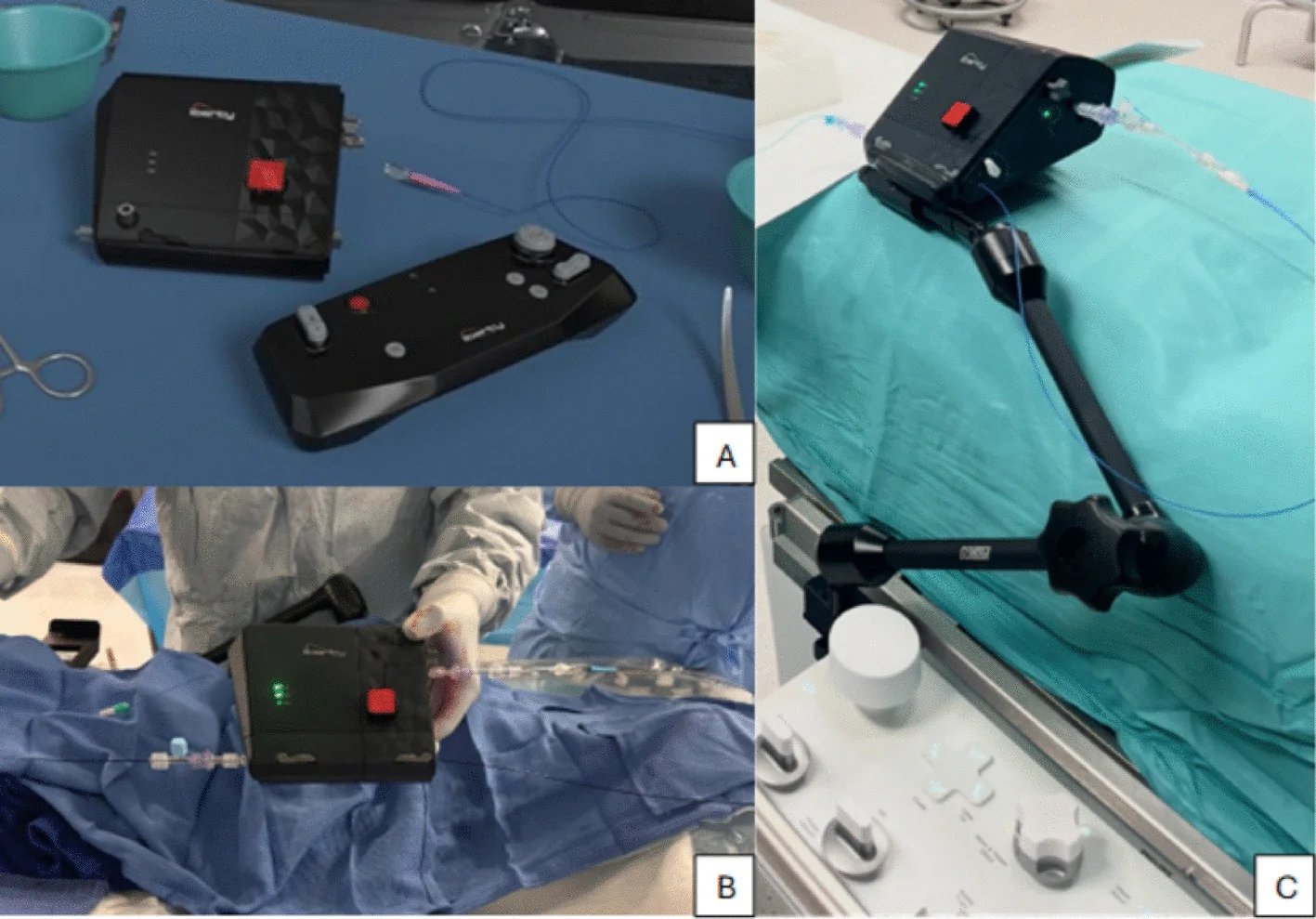
LIBERTY® Endovascular Robotic System angiosuite set-up

As illustrated in Fig. 2, the compact robotic drive is positioned within the sterile field at the patient’s access site and mounted to the procedure table. Once the base catheter is in appropriate positioning, the operator loads a microcatheter and microwire into the single-use robotic drive, then uses a handheld wireless controller and real-time fluoroscopic imaging to remotely select target vessels. In our institutional experience, it takes approximately 5 min to set up the robot during the procedure. This setup allows for incorporation of new robotic technology while maintaining a standard angiosuite workflow with minimal additional equipment or room reconfiguration, roughly at the added expense of a specialty microcatheter. Given its wireless control capabilities, the system also has the potential to support telerobotic operation. This study describes our institution’s experience with the first interventional procedures with the PEDRA after recent FDA approval and describes the first documented utilization of PEDRA for PAE, UFE, renal/gonadal venography, GAE, and Y90 radioembolization, as well as the first documented utilization of robotic assistance from left radial access.

## Results

### Demographics and procedural breakdown

Patient demographics are presented in Table 1. The mean patient age in years was 66.5 with a standard deviation of 15.5. Cardiovascular disorders including history of hypertension or diabetes were present in 45% (9/20) of patients. Participants were 65% male (13/20) and 35% female (7/20). Patient race was mixed, including 55% (11/20) White, 30% (6/20) Black or African American, 5% (1/20) Asian, and 10% (2/20) Other.

**Table 1:** Patient demographic characteristics

**Characteristic**	Value (*n* = 20)
Age, years, mean ± SD	66.5 ± 15.5
Male sex, *n* (%)	13 (65%)
Female sex, *n* (%)	7 (35%)
Diabetes mellitus, *n* (%)	2 (10%)
Hypertension, *n* (%)	9 (45%)
**Ethnicity, *n* (%)**	
Hispanic or Latino	1 (5.0%)
Non-Hispanic or Latino	19 (95%)
**Race, *n* (%)**	
American Indian or Alaska Native	0 (0.0%)
Asian	1 (5.0%)
Black or African American	6 (30%)
Native Hawaiian or other Pacific Islander	0 (0.0%)
White	11 (55%)
Other	2 (10%)

Of the 20 procedures completed, 12 were PAE, 1 was a UFE, 1 was a renal/gonadal venography, 2 were GAE, 2 were selective hepatic angiography for Y90 mapping, and 2 were Y90 radioembolization of a hepatic tumor. Access points included 15 left radial artery, 2 right femoral artery, 2 left femoral artery, and 1 right femoral vein, all of which were decided based on individual procedural plans and patient characteristics.

#### Procedural technical success

Success metrics are outlined in Table 2. There was 100% (20/20) procedural technical success in all endovascular procedures performed.

**Table 2:** Outcome metrics

**Technical success, *n* (%)**	20/20 (100%)
**Complete robotic target-vessel selection, *n* (%)** **Total robotic attempted vessels selected, *n* (%)**	15/20 (75%) 31/31 (100%)
**Partial robotic target-vessel selection, *n* (%)**	5/20 (25%)
Unsuccessful robotic selection, *n* (%)	0/5 (0%)
Robotic malfunction, *n* (%)	5/5 (100%)
**Device-related adverse events, *n* (%)**	0 (0.0%)
**Procedural complications, *n* (%)**	0 (0.0%)
**Subgroup analysis, *n* (%)**	
**Initial robotic units, cases 1–10**^**†**^	
Complete robotic target-vessel selection, *n* (%)	5/10 (50%)
Robotic malfunction, *n* (%)	5/10 (50%)
**Updated robotic units, cases 11–20**^**††**^	
Complete robotic target-vessel selection, *n* (%)	10/10 (100%)

^†^ Initial robotic units; oversensitive fail-safe led to error mode in 5/10 cases, with conversion to manual

^††^ Updated robotic units; no robotic malfunctions observed in subsequent 10/10 cases

#### Complete robotic target-vessel selection

There was 75% (15/20) complete robotic target-vessel selection (CRTVS) when utilizing PEDRA in these interventions. Subgroup analysis of the last 10 cases after utilizing the second generation LIBERTY® robot had 100% (10/10) CRTVS.

#### Total robotic attempted vessels selected

Within the 20 cases performed with PEDRA, all 31 vessels attempted with robotic assistance were successfully selected.

#### Partial robotic target-vessel selection—unsuccessful robotic selection

There were no cases of partial robotic target-vessel selection (PRTVS) secondary to unsuccessful robotic selection. All cases of PRTVS had initially successful target vessel selections but resulted in continuation of the case manually due to robotic malfunction.

#### Partial robotic target-vessel selection—robotic malfunction

There was 25% (5/20) PRTVS secondary to robotic malfunction. Of note, these 5 cases were all within the first 10 cases performed with the first produced units. The subsequent 10 cases were performed with updated robotic units, which had no incidence of robotic malfunction. The robotic malfunction of mention was a fail-safe programmed in the first produced units of the robot, encountered when utilizing “turbo mode” to advance and/or withdraw the microwire quickly when needed, which was oversensitive to friction forces. This oversensitive fail-safe led to an “error mode” in several cases where the robot could not be used for the remainder of the case, leading to conversion to manual selection.

#### Procedure time and radiation dose per procedure

Below you will see the individual procedural times as well as radiation doses for each (Table 3).

**Table 3:** Radiation dose per procedure

Case number	Total procedural time (min)	Fluoro time (min)	Total DAP (Gy-cm^3^)	Total air kerma (mGy)	Complete or partial robotic target-vessel selection
1—PAE	187.6	6.8	38.6	144	Complete
2—PAE	200.4	20.5	155	680	Partial
3—PAE	200	64	255	2429	Complete
4—PAE	80.6	15.2	295	930	Partial
5—Y90 Mapping	94.7	32	14.9	58.5	Complete
6—PAE	168	46.3	875	5109.24	Complete
7—Y90 Mapping	64.9	8.4	88	298	Complete
8—Y90 Treatment	156.7	49.9	512	2482	Complete
9—GAE	162.5	49.8	336	1758	Complete
10—GAE	170.8	64	64	442	Partial
11—PAE	132.8	41.3	262	1016	Partial
12—PAE	155.5	24.1	344	1541	Complete
13—Y90 Treatment	86.58	21.9	17.5	64.4	Complete
14—PAE	131.97	40.3	302	1069	Complete
15—PAE	161.67	42.8	198	1029	Complete
16—PAE	145.62	56	159	725	Partial
17—UFE	289.13	31.1	135	587.56	Complete
18—Left renal/gonadal venography	53.98	8.5	130	405	Complete
19—PAE	130.22	34.9	294	1499	Complete
20—PAE	138.22	23	238	847	Complete
Average	145.59	34.04	236	1155.69	

Two cases had total air kerma of 2 Gy–5 Gy and one case was above 5 Gy in radiation dose. None of these cases were associated with PRTVS or robotic malfunction. Upon retrospective review, these patients underwent prostate artery embolization and had high BMI with tortuous iliac arterial anatomy. Patients at our institution who receive over 2 Gy of radiation dose are counseled on potential for skin erythema or injury and followed up in clinic for further evaluation. The average procedural time for CRTVS compared to PRTVS respectively was 145.44 min vs. 146.04. The average DAP for CRTVS compared to PRTVS respectively was 251.87 Gy cm^3^ vs. 187 Gy cm^3^.

#### Adverse events

There were no device-related or procedural serious adverse events (SAE) or complications in any of the 20 procedures.

#### Devices utilized

All base catheters, microcatheters, and microwires utilized with the PEDRA are listed (Table 4). Base catheters for all procedures were 5 F, ranging in length from 80 to 135 cm. Microcatheters were 1.9F–2.8F, ranging in lengths from 150 to 175 cm. Microwires utilized were 0.014″–0.016″ and ranging in lengths from 220 to 300 cm.

**Table 4:** Procedural data and device compatibility

Case number	Procedure	Access	Catheter(s)	Microcatheter(s)	Microwire(s)	Embolic/material	CBCT used	Target vessels
1	PAE	Left radial	5 F 125 cm RAVI MG1	2.0F 175 cm Truselect	300 cm 0.014″ Runthrough	n-BCA	Yes	Left prostatic artery, right prostatic artery
2	PAE	Left radial	5 F 135 cm RAVI MG2	2.2F 165 cm Sniper	300 cm 0.014″ Runthrough	n-BCA	Yes	Left prostatic artery
3	PAE	Left radial	5 F 125 cm RAVI MG1	2.0F 175 cm Truselect	300 cm 0.014″ Runthrough	n-BCA	Yes	Left prostatic artery, right prostatic artery
4	PAE	Left radial	5 F 125 cm RAVI MG1	2.0F 175 cm Truselect	300 cm 0.014″ Runthrough	n-BCA	Yes	Left prostatic artery
5	Y-90 Mapping	Left CFA	5 F SOS 2	2.0F 175 cm Truselect	300 cm 0.014″ Runthrough	MAA	Yes	Right hepatic artery
6	PAE	Left radial	5 F 135 cm RAVI MG2	2.0F 175 cm Truselect	300 cm 0.014″ Runthrough	Particles	Yes	Left prostatic artery, right prostatic artery
7	Y-90 Mapping	Left radial	5 F 125 cm RAVI MG1	2.0F 175 cm Truselect	300 cm 0.014″ Runthrough	MAA	Yes	Right hepatic artery
8	Y90 Treatment	Left radial	5 F 125 cm RAVI MG1	2.0F 175 cm Truselect	300 cm 0.014″ Runthrough	Theraspheres	No	Right hepatic artery
9	GAE	Left CFA	5 F 100 cm GlideCatheter	2.4F 150 cm Progreat	220 cm 0.016″ GT-R	Lipiodol	No	Right descending genicular artery, right superior lateral genicular artery,
10	GAE	Right CFA	5 F 125 cm vertebral catheter	2.0F 175 cm Truselect	300 cm 0.014″ Synchro Soft	Imipenem/Cilastatin	No	Left descending genicular artery, left lateral genicular artery
11	PAE	Right CFA	5 F 100 cm RUC	2.0F 150 cm Truselect	300 cm 0.014″ Synchro Soft	Particles	No	Left prostatic artery
12	PAE	Left radial	5 F 125 cm RAVI MG1	1.9F 165 cm Progreat Lambda Straight Tip	220 cm 0.016″ GT-R	n-BCA	Yes	Left prostatic artery, right prostatic artery
13	Y-90 Treatment	Left radial	5 F 125 cm RAVI MG1	2.0F 175 cm Truselect	300 cm 0.014″ Runthrough	SIR-Spheres	Yes	Right hepatic artery
14	PAE	Left radial	5 F 135 cm RAVI MG2	2.0F 175 cm Truselect	300 cm 0.014″ Runthrough	n-BCA	Yes	Left prostatic artery, right prostatic artery
15	PAE	Left radial	5 F 125 cm RAVI MG1, 5 F 125 cm Vanschie3	2.0F 175 cm Truselect	300 cm 0.014″ Runthrough	n-BCA	Yes	Left prostatic artery, right prostatic artery
16	PAE	Left radial	5 F 125 cm RAVI MG1, 150 cm Cobra2	2.0F 175 cm Truselect	300 cm 0.014″ Runthrough	n-BCA	Yes	Left prostatic artery
17	UFE	Left radial	5 F 125 cm vertebral catheter	2.8F 165 cm Progreat	300 cm 0.014″ Fathom	Particles	No	Left uterine artery, right uterine artery
18	Left renal/gonadal venography	Right CFV	5 F 80 cm SOS2	2.4F 150 cm Progreat	300 cm 0.014″ Fathom	None	No	Left gonadal vein
19	PAE	Left radial	5 F 125 cm RAVI MG1	2.0F 175 cm Truselect	300 cm 0.014″ Runthrough	n-BCA	Yes	Left prostatic artery, right prostatic artery
20	PAE	Left radial	5 F 125 cm RAVI MG1	2.0F 175 cm Truselect/2.0F 165 cm Progreat	300 cm 0.014″ Runthrough	n-BCA	Yes	Left prostatic artery, right prostatic artery

## Discussion

Our early experience demonstrates that PEDRA integrates into diverse interventional radiology practices with high technical success and safety, requiring minimal workflow modification. Our use of PEDRA with PAE, UFE, renal/gonadal venography, GAE, Y90 radioembolization, and radial access is novel to robotic-assisted endovascular literature and highlights the system's versatility.

As with early adoptions of laparoscopic or CT-guided robotic interventions, measurable improvements over traditional techniques are initially obscured by the learning curve [8–10]. While the heterogeneity of operators and procedure types makes the fluoroscopy dose and procedure time difficult to interpret, we observed expected initial increases in setup time as operators prioritized device familiarity. One of the novelties of the PEDRA is the use of haptic feedback. In procedural robotics, this has been a technical shortcoming for years due to the complexity of translating mechanical force over a distance in a low latency manner to provide real-time feedback to an operator [11]. The value of such cannot be understated. Touch is an essential component of endovascular procedures, and the absence of haptic feedback has historically been a barrier to robotic adoption due to concerns about unintentional vascular injury [12, 13]. Delivering on this capability addresses that concern and smooths workflow integration, as it allows operators to approach these procedures more closely aligned with a manual method: without losing their sense of touch.

Theoretically, PEDRA enables remote subselection of vasculature from a control room, potentially eliminating the need for lead protection and reducing both mechanical strain and radiation exposure to the physician operator. We anticipate that structured training will eventually yield significant ergonomic and safety benefits for the primary operator over conventional methods. Nevertheless, implementation of such technology, especially remotely, would continue to require trained personnel such as advanced practice providers (APPs), trainees, or other physicians to be with the patient to assist with preparation, vascular access, initial base catheter manipulation, and docking of the robotic system while the primary operator performs the most critical portions of the procedure remotely. This correlates to an on-site APP or technician assisting with initial access and docking of robotic arms prior to remote robotic surgery. Even as future iterations of the technology allow for more advanced techniques (contrast injection, base catheter manipulation, etc.) in the hands of the primary operator, we suspect that a trained technologist will be at minimum needed on-site to assist with aspects of the procedure. Unlike previous endovascular robots, PEDRA’s portable, disposable nature lowers economic and institutional barriers to adoption. Its compact footprint eliminates the need for large base consoles or significant capital investment, making the technology feasible for rural settings and non-academic centers. This small form factor also facilitates easier integration for support staff and radiology technologists, as the system is single use, requires minimal setup, and does not necessitate dedicated room reconfiguration, capital equipment maintenance, or specialized technical training. Furthermore, PEDRA exemplified the first endovascular robot to employ haptic feedback into the procedure, a feat not met in previous endovascular robotic designs. It was also compatible with a greater breadth of wires and did not necessitate large infrastructural requirements compared to its predecessors. PEDRA’s potential for telehealth applications could expand access to specialty minimally invasive care, reducing costs and delays associated with patient transfers, or offering care not otherwise available in rural areas [14]. As this new technology continues to develop and institutions grow with the learning curve, there is a potential role for implementation of robotic endovascular technology specifically at institutions with lower procedural volumes, such as community hospitals or pediatric interventional radiology services [15].

Based on our institutional experience overcoming the initial learning curve, we offer the following technical considerations to optimize procedural efficiency:

When thinking about catheter, microcatheter, and microwire compatibility with the PEDRA, a general rule of thumb that we noticed is the “50–50” rule; after choosing a base catheter that is best suited for the planned intervention, the microcatheter should be at least 50 cm longer than the length of the base catheter, and then the microwire should be at least 50 cm longer than the microcatheter. Following this rule is an easy way to avoid running out of length when maneuvering during the procedure. With this being said, a 300 cm 0.014″–0.016″ microwire can be used with any base catheter and microcatheter combination to avoid having any technical issues with compatibility and length.

In our PAE practice, the robot is introduced only after the base catheter reaches the internal iliac artery and initial imaging, including cone beam computerized tomography (CBCT) or ipsilateral oblique digital subtraction angiography (DSA), has been completed. We utilize embolization guidance software with CBCT to optimize the oblique angle, followed by a confirmatory DSA with the table locked. For left radial access, the robotic side arm is connected to the ipsilateral rail near the wrist. Proper positioning ensures the robot remains clear of the C-arm’s swing during CBCT. Equipment selection follows a “short base/long microcatheter” strategy. Our standard setup includes a 5 F 125–135 cm RAVI MG1/MG2 base catheter (Terumo Medical Corp, Somerset, NJ, USA), a 175 cm 2.0F TRUSELECT microcatheter (Boston Scientific, Marlborough, MA, USA), and a 300 cm 0.014″ Runthrough wire (Terumo Medical Corp, Somerset, NJ, USA). While smaller microcatheters may be used in PAE, the robot’s IFU specifies compatibility with the 2.0–3.0F range. A torque is placed on the wire end to maintain sterility and prevent the wire from entering the gearbox. A blue towel is draped over the knob/rail system to prevent microcatheter snagging.

To perform subsequent DSAs or reshape the microwire tip, the wire is retracted into the microcatheter robotically, then physically removed and placed on the sterile drape. In challenging anatomy, such as Type 1 prostatic arteries, the robot can be disconnected to facilitate a base catheter exchange (e.g., to a 5 F Van Schie 3). After re-establishing position in the vesicular branch, the robot is reconnected, and robotic-assisted sub-selection may resume.

Postembolization, the microcatheter is vigorously flushed and inspected extracorporeally before reinsertion. For contralateral treatment, the base catheter is advanced to the opposite internal iliac artery, imaging is repeated, and the robot is reset to complete the procedure.

From our experience utilizing PEDRA for hepatic mapping angiography, the robot is introduced at the same time as the microcatheter and microwire. In our practice, we often use the 125 cm 5 F RAVI MG1 base catheter (Terumo Medical Corp, Somerset, NJ, USA) with a left radial artery approach. An alternative is a 110 cm 5 F Sarah Radial OPTITORQUE base catheter (Terumo Medical Corp, Somerset, NJ, USA). The microcatheter and microwire combination can be based on operator preference. Frequently used combinations include a 150 cm 2.4F or 2.8F Progreat microcatheter (Terumo Medical Corp, Somerset, NJ, USA) or a 175 cm 2.0F TRUSELECT microcatheter (Boston Scientific, Marlborough, MA, USA). A 220 cm GLIDEWIRE GT-R or 300 cm Runthrough (Terumo Medical Corp, Somerset, NJ, USA) has typically been utilized. Of note, in scenarios where there is a distal subselective lesion, the 125 cm base catheter and 150 cm microcatheter do not leave as much working room when utilizing the robot. We typically will use a 175 cm microcatheter with a 125 cm base catheter or the 150 cm microcatheter with the 110 cm base catheter.

The robot is then set up as described above for the left radial PAE. The side arm is connected ipsilateral, and the robot is placed where CBCT can still be utilized. It is our practice to use CBCT to determine all vessel feeders to tumor using embolization guidance. A separate CBCT is performed at the intended point of therapy with Y90 radioembolization to determine the “angiosome” supplied by the vessel. 99Tc-MAA has been injected through the microcatheter while in the robot without difficulty. Given the radiopharmaceutical is in the lumen of the microcatheter and not on the outside, the microcatheter is disposed of in nuclear waste, but the robot can be disposed of normally.

When using PEDRA for Y90 radioembolization treatment, all of the principles described in the section regarding hepatic mapping angiography above are also utilized. There is one technical note—our operators typically use the same microcatheter type for mapping and delivery of Y90 radioembolization (to ensure mapping and delivery are similar). In this regard, the choice of microcatheter may be determined by the radioembolization device utilized. Glass microspheres have been injected safely through the 2.0F microcatheters in the robot. Resin microspheres tend to warrant use of a 2.8F microcatheter. Lengths are then planned out appropriately. In our experience, Y90 has been delivered through the microcatheter while in the robot without difficulty. The microcatheter can be disposed of in nuclear waste. The robot can be disposed of normally. The operators advise disabling the robot and unlocking the gears before Y90 is delivered—this is to ensure there are no mild “pinch points” that could impede Y90 microsphere forward flow.

From our limited experience with performing robotic-assisted GAE, a 5 F 100 or 125 cm base catheter is typically sufficient for up and over access, from the contralateral common femoral artery, to treat geniculate artery branches of the distal superficial femoral artery (SFA) and popliteal artery. Our experience with the PEDRA was to use a microcatheter about 50 cm longer than the base catheter, which was why we used a 2.4F 150 cm Progreat with the 100 cm Glidecatheter (Terumo Medical Corp, Somerset, NJ, USA), and a 2.0F 175 cm TruSelect (Boston Scientific, Marlborough, MA, USA) with the 125 cm vertebral catheter (Merit Medical, Jordan, UT, USA). If a good power-injected DSA is performed from the distal SFA, it is likely that multiple geniculate artery branches can be selected with PEDRA utilizing the same initial roadmap. Otherwise, additional DSA can be performed, especially if an oblique is needed to better visualize a vessel origin or to avoid obscuration by an arthroplasty hardware, but this would require partial disassembly of the catheter, microcatheter, and microwire unit from the PEDRA. Access for this procedure can also be done antegrade from the ipsilateral common femoral artery or retrograde from the ipsilateral pedal artery. However, based on our experience with the PEDRA setup, we found a contralateral femoral approach to be the most ergonomic configuration for robotic assistance due to the physical orientation and positioning of the robotic device. Specifically, an ipsilateral antegrade femoral approach would require the robotic system to be oriented distally down the ipsilateral leg, creating a less favorable working angle and more cumbersome device setup compared with approaching from the contralateral side.

As seen by the subgroup analysis of our institution's first 10 cases with the initial production of the robot when compared to the subsequent 10 cases with updated units which accounted for robotic malfunction due to over-sensitivity to friction forces, there will continue to be advances in this technology which will continue to improve efficiency, efficacy and maneuverability with increasing reliability without robotic malfunction. Future models will plan to incorporate features allowing for contrast injection and embolization with the robot in place. Endovascular robotic utility extends beyond radiation reduction. These systems may help standardize procedural execution across operators, potentially reducing variability between trainees and experienced interventionalists while improving consistency across institutions and geographic regions. This democratization of technical capability may have implications not only for procedural reproducibility but also for broader access to complex endovascular interventions [16].

## Conclusion

In this single-center, retrospective series of 20 consecutive outpatient interventions, the PEDRA demonstrated feasible integration across diverse procedures and vascular access sites with 100% procedural technical success and a 75% complete robotic target-vessel selection with 100% robotic target-vessel selection when evaluating the last 10 procedures performed with updated units and all 31 attempted vessels successfully selected with robotic assistance (100%). There were no device-related serious adverse events. The system’s compact, disposable architecture enabled left radial and bilateral femoral approaches and novel applications including PAE, UFE, renal/gonadal venography, GAE, and Y90 radioembolization, suggesting potential to lower infrastructural and economic barriers to broader adoption when compared to previous endovascular robotic systems. Limitations include small sample size, single-center retrospective design, operator learning curve, and lack of standardized radiation and procedural time measurements. Prospective multicenter studies with standardized radiation monitoring, ergonomic and learning-curve assessments, cost analyses, and procedure-specific outcomes are warranted to validate these early findings and define PEDRA’s role in routine endovascular practice.

## Supplementary Information

Additional file 1.

## Data Availability

All data generated or analyzed during this study are included in this published article.

## Abbreviations

PEDRA: Portable endovascular disposable robotic assistant
IRB: Institutional Review Board
PAE: Prostate artery embolization
GAE: Geniculate artery embolization
CRTVS: Complete robotic target-vessel selection
PRTVS: Partial robotic target-vessel selection
SAE: Serious adverse events
CBCT: Cone beam computerized tomography
DSA: Digital subtraction angiography
SFA: Superficial femoral artery
